# miR-139-5p protects septic mice with acute lung injury by inhibiting Toll-like receptor 4/Myeloid differentiation factor 88/Nuclear factor-&mac_kgr;B signaling pathway

**DOI:** 10.6061/clinics/2021/e2484

**Published:** 2021-03-01

**Authors:** Xiuxiu Zhang, Xin Liu, Rui Chang, Yue Li

**Affiliations:** I Departments of Critical Care Medicine, Eastern District of the Ji’ning No.1 People’s Hospital, Ji’ning, Shandong 272000, P.R. China; II Departments of Emergency Critical Care Medicine, Eastern District of the Ji’ning No.1 People’s Hospital, Ji’ning, Shandong 272000, P.R. China

**Keywords:** miR-139-5p, Sepsis, Acute Lung Injury

## Abstract

**OBJECTIVES:**

To investigate the role of miR-139-5p and the TLR4/MyD88/NF-κB signaling pathway in acute lung injury in septic mice.

**METHOD:**

A total of 140 healthy male SPF C57BL/6 mice were divided into seven groups, *i.e.*, Normal, Control, NC, miR-139-5p mimic, miR-139-5p inhibitor, TAK-242, and miR-139-5p inhibitor+TAK-242 groups. The levels of miR-139-5p, proteins related to the TLR4/MyD88/NF-κB signaling pathway (TLR4, MyD88, and p-NF-κB p50), and MPO, SOD, GSH, and MDA in lung tissue were measured. The lung tissue wet-to-dry mass ratio (W/D), arterial oxygen partial pressure (PaO_2_), and carbon dioxide partial pressure (PaCO_2_) were measured.

**RESULTS:**

A web-based bioinformatic tool predicted that MyD88 was a target of miR-139-5p, which was verified by a dual luciferase reporter assay. Compared with those in the Normal group, the levels of miR-139-5p, PaO_2_, SOD, and GSH were significantly lower, while those of TLR4, MyD88, p-NF-κB p50, W/D, PaCO_2_, IL-1β, TNF-α, IL-6, MPO, and MDA were higher in all other groups. Moreover, compared with their levels in the Control group, these indicators exhibited contrasting results in the miR-139-5p mimic and TAK-242 groups, but were similar in the miR-139-5p inhibitor group. In the miR-139-5p inhibitor+TAK-242 group, acute lung injury, aggravated by miR-139-5p inhibitor, was partially rescued by TAK-242.

**CONCLUSION:**

miR-139-5p inhibits the TLR4/MyD88/NF-κB signaling pathway to alleviate acute lung injury in septic mice.

## INTRODUCTION

Sepsis is the most common cause of acute lung injury (ALI) and acute respiratory distress syndrome (ARDS), which are developed from the chain reaction of collective inflammation triggered by the occurrence of sepsis (1). ALI is characterized by extensive inflammation and life-threatening hypoxemia caused by differences in ventilation and perfusion in the lungs (2). ARDS, also known as shock lung in the past, is caused by trauma or pulmonary infection, particularly sepsis. Importantly, the risk of developing ARDS is higher when there are multiple risk factors for ALI (3). Combined activation of coagulation and inflammation contributes to multiple organ dysfunction and a poor prognosis after severe trauma (4,5). A previous study showed that the combined activation of platelets and leukocytes is associated with the severity of organ dysfunction in sepsis (6). In addition, systemic activation of inflammation and coagulation associated with endothelial damage has prognostic value for the development of ALI/ARDS (6).

microRNAs (miRNAs) are endogenous small non-coding RNAs that regulate the expression of protein-coding or non-coding genes in a sequence-specific manner (7). miRNAs regulate numerous cellular processes, including development, apoptosis, differentiation, metabolism, and stress responses, depending on the regulation of specific target genes (8). Abnormal expression of miR-139-5p is frequently observed in human diseases, such as congenital heart disease and chronic myeloid leukemia (9-11). Moreover, miR-139-5p is known to play a role in bladder cancer, laryngeal squamous cell carcinoma, and breast cancer (12-14). However, the function of miR-139-5p has not been studied in ALI, and the relationship between miR-139-5p and inflammation has not been elucidated.

Numerous studies depict the important role of TLRs in the innate immune system, which is responsible for inflammation in ALI. The TLR family comprises 10 members (TLR1-10), among which TLR4 initiates a series of responses, including neutrophil infiltration and the accumulation of cytokines (15). After binding to TLR4 via LPS, it sends signals into the cells to activate the IRAK of downstream molecule, MyD88. Then, IRAK4 and IRAK1 are recruited to the MyD88 complex, which in turn leads to the activation of IKKα and IKK-β, and the NF-κB, a regulator of inflammatory diseases, induces the transcription of multiple cytokines, including IL-6, IL-1β, and TNF-α (16,17). Therefore, TLR4 plays a key role in NF-κB activation and in the pathogenesis of various lung diseases, such as ALI (18).

In our study, we identified MyD88 to be a target of miR-139-5p through bioinformatic prediction. We also found that MyD88 was downregulated in ALI. Therefore, we speculate that miR-139-5p targets and downregulates MyD88 expression, thereby inhibiting the MyD88/NF-κB signaling pathway to relieve ALI in septic mice.

## MATERIALS AND METHODS

### Animals and grouping

This study included 140 healthy specific pathogen free male C57BL/6 mice, with a clean grade and body weights of 35±5 g. The ‘Normal’ group comprised 18 randomly selected mice, while the remaining mice were used to establish the sepsis model. These sepsis model mice were divided into the following 6 groups: control group (without treatment), NC group (injected with negative control of miR-139-5p), miR-139-5p mimic group (injected with an miR-139-5p overexpression vector), miR-139-5p inhibitor group (injected with a miR-139-5p silencing vector), TAK-242 group (injected with a TLR4 inhibitor), miR-139-5p inhibitor+TAK-242 group (combination treatment), with 15 mice per group. The experiment was repeated three times, with the above number of mice inclusive of repeated experiments. The adenovirus vectors harboring the NC sequence, miR-139-5p mimic sequence, or miR-139-5p silence sequence (synthesized by Shanghai Jima Co., Ltd., China), were supplied by Tianjin Xaer Biotechnology Co., Ltd., China. The treatment dosage of the virus was 20 mg.kg^-1^. day^-1^. The TAK-242 stock solution (AbMole, USA, 10 mg/mL) was diluted with 1×PBS and administered through the tail vein at a dose of 10 mg/kg once a week for 3 weeks.

Subsequently, the sepsis model was established using the cecal ligation and puncture method. Briefly, the mice were fixed on the operating table and anesthetized by intraperitoneal injection of 3% pentobarbital sodium (50 mg/kg; Shanghai Qibai Biotechnology Co., Ltd.). A 1 cm incision was made in the central part of the anterior abdomen of the mouse to open the intestinal membrane and expose the cecum end. Then, the root of the cecum was ligated, and a 27G needle was punctured into the cecum. Next, the cecal contents were extruded, and the cecum was sutured. Pre-warmed saline was intravenously injected postoperat ively. Thirty-two animals died, and the success rate of the establishment of the model was 73.77%. In each experiment, lung tissue and venous blood were collected from 5 mice in each group. Some lung tissues were fixed in 10% neutral formalin (Shanghai Qibai Biotechnology Co., Ltd.) for 24h, dehydrated in alcohol, and paraffinized. The remaining tissues were stored in liquid nitrogen. This study was approved by the Animal Care and Use Committee of the Eastern District of the Jining No.1 People's Hospital Ji'ning, China.

### Dual luciferase reporter system

The miR-139-5p binding site in MyD88 was predicted using TargetScan (http://www.targetscan.org). The relationship between miR-139-5p and MyD88 was verified using the dual luciferase reporter system. The target gene dual luciferase reporter vector and the mutants that bind to the miR-139-5p binding site were constructed separately as PGL3-MyD88wt and PGL3-MyD88mut. Then, 4 groups of HEK293T cell line were treated with the following: Rellina plasmid, PGL3-MyD88wt, and miR-139-5p plasmid; Rellina plasmid, PGL3-MyD88wt, and NC plasmid; Rellina plasmid, PGL3-MyD88mut, and miR-139-5p plasmid; Rellina plasmid, PGL3-MyD88mut, and NC plasmid. After 24h of cell transfection, the dual luciferase reporter assay was performed to measure luciferase activity in accordance with the manufacturer’s (Promega) guidelines. Luciferase activity was calculated using the following equation: Relative luciferase activity=firefly luciferase/Renilla luciferase (19).

### qRT-PCR

Total RNA was extracted using TRIzol (Cat. No. 16096020, Thermo Fisher Scientific, New York, USA). Complimentary DNA was synthesized by reverse transcription using TaqMan MicroRNA Assay Reverse Transcription Primers (Thermo Scientific, USA). The SYBR^¯^ PremixExTaq^TM^ II Kit (Xingzhi Biotechnology Co., Ltd., China) was used for quantitative PCR. The following components were added in sequence: 25 μL LBRBR PremixExTaq^TM^ II (2×), 2 μL PCR forward and reverse primers, 1 μL ROX Reference Dye (50×), 4 μL DNA template, and 16 μL ddH_2_O. Quantitative PCR was performed on ABIPRISM^¯^ 7300 (model Prism^¯^ 7300, Shanghai Kunke Instrument Equipment Co., Ltd., China). The reaction conditions were: pre-denaturation at 95°C for 10 min, denaturation at 95°C for 15s, annealing at 60°C for 30s, for 32 cycles, extension at 72°C for 1 min. For evaluating the expression of miR-139-5p, U6 was used as an internal reference, while for other genes, GAPDH was used as an internal reference gene. The relative expression of each gene of interest was calculated using the 2^-ΔΔCt^ metho d. The primers used are shown in Table 1. 

### Western blot

Total protein was extracted using RPPA lysis buffer (R0010, Solarbio) containing PMSF, and protein concentration was determined using a BCA kit (Thermo, USA). The sample was denatured by heating in the loading buffer at 100°C for 10 min. Then, 50 μg of protein was loaded, electrophoresed at 70 V for 3h, and transferred onto a PVDF membrane (ISEQ00010, Millipore, Billerica, MA, USA) at a constant flow of 150 mA. The membrane was blocked with 5% skim milk (Shanghai Xinyu Biotechnology Co., Ltd.) at room temperature for 4h at 20°C; washed with TBST; incubated with rabbit antibodies of TLR4 (ab13556, 1:500, Abcam, UK), MyD88 (ab2064, 1:500, Abcam, UK), NF-κB p50 (ab220803, 1 µg/mL, Abcam, UK), p-NF-κB p50 (phospho S337) (ab28849, 1 μg/mL, Abcam, UK), and GAPDH (ab22555, 1:2,000, Abcam, UK) overnight at 4°C; and washed with TBST again. Subsequently, the membrane was incubated with HRP-labeled goat anti-rabbit IgG antibody (Beijing Zhongshan Biotechnology Co., Ltd., diluted 1:5,000) for 2h, washed with TBST, and developed using the ECL fluorescence detection kit (Cat. No. BB-3501, Amersham, UK) on a Bio-Rad image analysis system (BIO-RAD, USA).

### Blood gas analysis and lung tissue wet/dry weight ratio

After the treatment and anesthetization by intraperitoneal injection of 3% pentobarbital sodium (50 mg/kg), the right hilar region of mice was blocked for 5 min and the carotid artery blood was collected for blood gas analysis. The observed indicators included arterial oxygen partial pressure (PaO_2_) and carbon dioxide partial pressure (PaCO_2_).

The left lung of each mouse was removed by thoracotomy, and was then blotted using filter paper to evaluate the lung wet weight. After drying in an 80°C incubator for 48h to a constant weight, the dry weight was estimated. The wet weight to dry weight ratio (W/D) and lung water content was calculated to reflect the degree of lung edema. The calculation used the following equation: Lung tissue W/D=(lung wet weight/lung dry weight)×100%.

### MPO activity

The myeloperoxidase (MPO) colorimetric activity assay kit (K744-100, Biovision, US) was used to measure MPO activity according to kit instructions. MPO activity unit was defined as the amount of the enzyme that catalyzes the degradation of 1 nmol of H_2_O_2_ per gram of tissue at 37°C. The activity was calculated as MPO=(absorbance of the experimental tube-absorbance of the control tube)/11.3×sample amount. MPO activity reflects the degree of neutrophil accumulation in the lung tissue.

### ELISA

Blood samples collected from mouse eyeballs were incubated at 4°C overnight, centrifuged at 3,500×g for 10 min, and the supernatant was collected and frozen at -80°C. The levels of IL-1β, TNF-α, and IL-6 were measured according to the ELISA test kit instructions. All kits were purchased from Wuhan Merck, China.

### Detection of SOD, GSH and MDA

The lung tissues (125 mm^3^) were homogenized in 1 mL of PBS, centrifuged at 12,000×g for 10 min at 4°C. The protein concentration in the supernatant was measured using the BCA test kit (P0011, Biyuntian), and malondialdehyde (MDA, A003-1-2), mitochondrial superoxide dismutase (SOD, A001-3-2), and reduced glutathione (GSH, A006-2-1) using kits from Nanjing Jiancheng Reagent Co., Ltd.

### Statistical analysis

All data were processed using SPSS (version 21.0) statistical software. Measurements were expressed as mean±standard deviation. One-way ANOVA and Tukey post hoc tests were used for comparisons between groups. Significance is indicated by *p*<0.05. 

## RESULTS

### miR-139-5p targets and negatively regulates the MyD88 gene in mouse lung tissue

**Figure 1:**
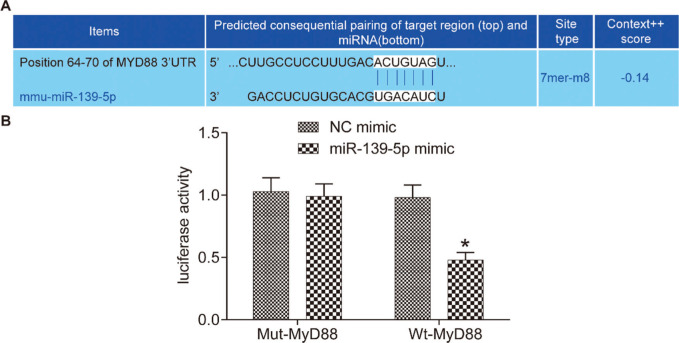
miR-139-5p targets MyD88 gene expression. The 3'-UTR region in which miR-139-5p binds to MyD88 (A); results of the dual luciferase reporter assay (B). The asterisk (*) indicates a significant difference compared with the NC group, where *p*<0.05. MyD88, myeloid differentiation factor 88.

The miR-139-5p binding site in MyD88 was predicted using a bioinformatic prediction website (http://www.targetscan.org) (Figure 1A). The results of the dual luciferase reporter assay verified this prediction (Figure 1B). Compared with the Wt-MyD88 plasmid+NC mimic group, the luciferase activity of the Wt-MyD88 plasmid+miR-139-5p mimic group was significantly lower (*p*<0.05). However, the luciferase activity of the Mut-MyD88 plasmid+miR-139-5p mimic group and Mut-MyD88 plasmid+NC mimic group did not show a significant difference (*p*>0.05). Therefore, miR-139-5p may target the negative regulation mechanism of the MyD88 gene.

### Expression of miR-139-5p and TLR4, MyD88 and p-NF-κB p50 in mice lung tissues

**Figure 2:**
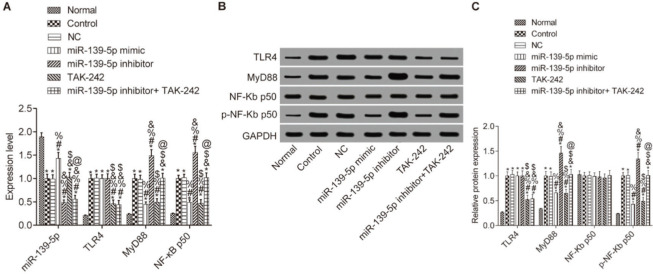
Expression of miR-139-5p, TLR4, MyD88, and NF-κB p50 in the lung tissue of each group. The mRNA levels of miR-139-5p, TLR4, MyD88, and NF-κB p50 in mouse lung tissue (A); The protein expression levels of TLR4, MyD88, NF-κB p50 and p-NF-κB p50 in mouse lung tissue (B-C). Significant comparisons with each group, where *p*<0.05, are indicated by: “*” for the Normal group, “#” for the Control group, “%” for NC group, “&” for the miR-139-5p mimic group, “$” for the miR-139-5p inhibitor group, and “@” for the TAK-242 group. TLR4, toll-like receptor 4; MyD88, myeloid differentiation factor 88.

We then determined whether miR-139-5p regulates TLR4/MyD88/NF-κB expression in mouse lung tissue (Figure 2). Compared with the Normal group, the expression levels of miR-139-5p in other groups were significantly downregulated, while the expression levels of TLR4, myD88, and p-NF-κB p50 were significantly upregulated (*p*<0.05), and the expression level of NF-κB p50 did not change (*p*>0.05). Compared with the Control group, the expression of MyD88 and NF-κB p50 was not significantly different in the NC group and the miR-139-5p inhibitor+TAK-242 group, while the expression of these molecules was significantly lower in the miR-139-5p mimic group and TAK-242 group, and significantly higher in the miR-139-5p inhibitor group (*p*<0.05). Compared with the miR-139-5p inhibitor group, the expression levels of MyD88 and p-NF-κB p50 were significantly lower in the miR-139-5p inhibitor+TAK-242 group (*p*<0.05). TLR4 expression was significantly lower in the TAK-242 group and the miR-139-5p inhibitor+TAK-242 g roup (*p*<0.05). There was a significant increase in miR-139-5p expression in the miR-139-5p mimic group, and a significant decrease in its expression in the miR-139-5p inhibitor group and miR-139-5p inhibitor+TAK-242 group as compared with NC group (*p*<0.05).

### The W/D, PaO_2_ and PaCO_2_ of mice in each group

**Figure 3:**
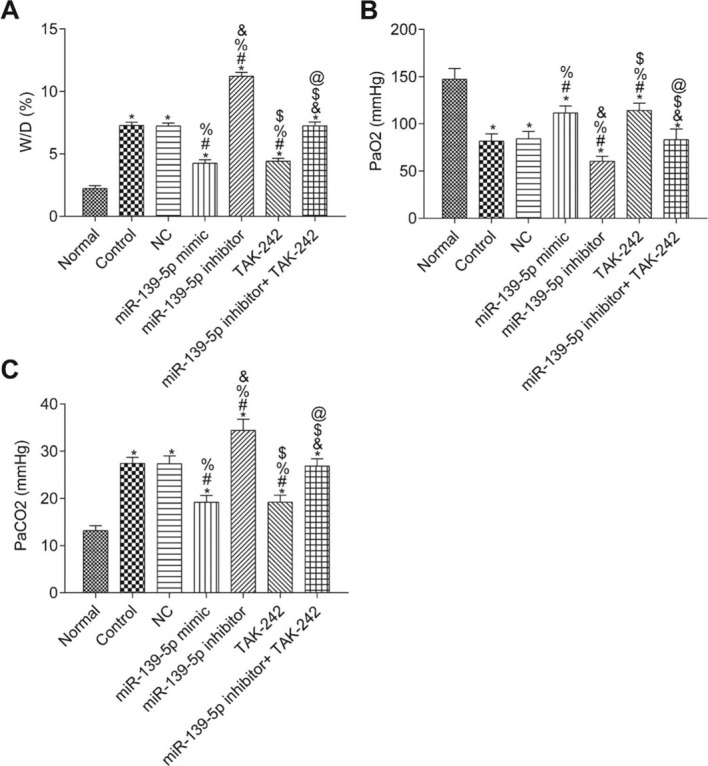
The W/D, PaO_2_ and PaCO_2_ of mice in each group. W/D of mouse lung tissue (A); PaO_2_ in mice (B); PaCO_2_ in mice (C). Significant comparisons with each group, where *p*<0.05, are indicated by: “*” for the Normal group, “#” for the Control group, “%” for NC group, “&” for the miR-139-5p mimic group, “$” for the miR-139-5p inhibitor group, and “@” for the TAK-242 g roup PaO_2_, arterial oxygen partial pressure; W/D, lung tissue wet-to-dry mass ratio; PaCO_2_, carbon dioxide partial pressure.

The W/D ratio and blood gas analysis results are shown in Figure 3. Compared with the Normal group, W/D and PaCO_2_ were significantly higher, but PaO_2_ was significantly lower in the other groups (*p*<0.05). Compared with the Control group, W/D, PaCO_2_, and PaO_2_ were not significantly different in the NC group and miR-139-5p inhibitor+TAK-242 group (*p*>0.05). Moreover, W/D and PaCO_2_ in the miR-139-5p mimic group and TAK-242 group were significantly lower, but PaO_2_ was significantly higher, compared to those of the Control group, and these indicators showed an opposite result in the miR-139-5p inhibitor group (all *p*<0.05). Compared with the miR-139-5p inhibitor group, W/D and PaCO_2_ were significantly lower, while PaO_2_ was significantly higher in the miR-139-5p inhibitor+TAK-242 group (*p*<0.05).

### Level of IL-1β, TNF-α and IL-6 in the serum of mice

**Figure 4:**
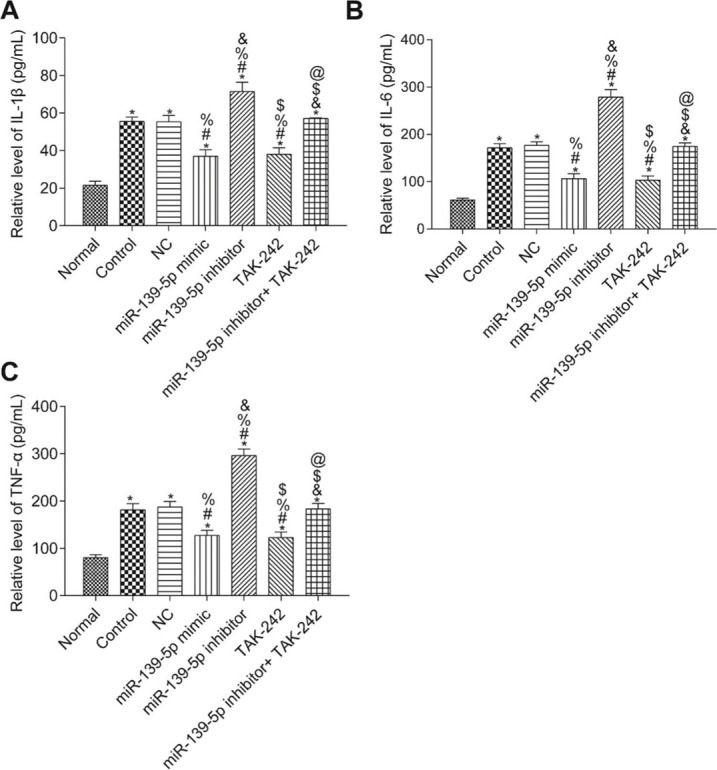
Serum levels of the inflammatory factors, IL-1β, IL-6, and TNF-α in mice of each group. IL-1β levels (A); IL-6 levels (B); TNF-α levels (C). Significant comparisons with each group, where *p*<0.05, are indicated by: “*” for the Normal group, “#” for the Control group, “%” for NC group, “&” for the miR-139-5p mimic group, “$” for the miR-139-5p inhibitor group, and “@” for the TAK-242 group.

The levels of IL-1β, TNF-α, and IL-6 in the serum of mice were measured by ELISA (Figure 4). Compared with those in the Normal group, the serum levels of IL-1β, TNF-α, and IL-6 were significantly higher in all other groups (*p*<0.05). Compared with those in the Control group, serum IL-1β, TNF-α, and IL-6 levels were not significantly different in the NC group and the miR-139-5p inhibitor+TAK-242 group (*p*>0.05), but were significantly lower in the miR-139-5p mimic group and TAK-242 group, and significantly higher in the miR-139-5p inhibitor group (*p*<0.05). Compared with those in the miR-139-5p inhibitor group, the serum levels of IL-1β, TNF-α, and IL-6 in the miR-139-5p inhibitor+TAK-242 group were significantly lower (*p*<0.05).

### Levels of MPO, SOD, GSH and MDA in lung tissues of mice in each group

**Figure 5:**
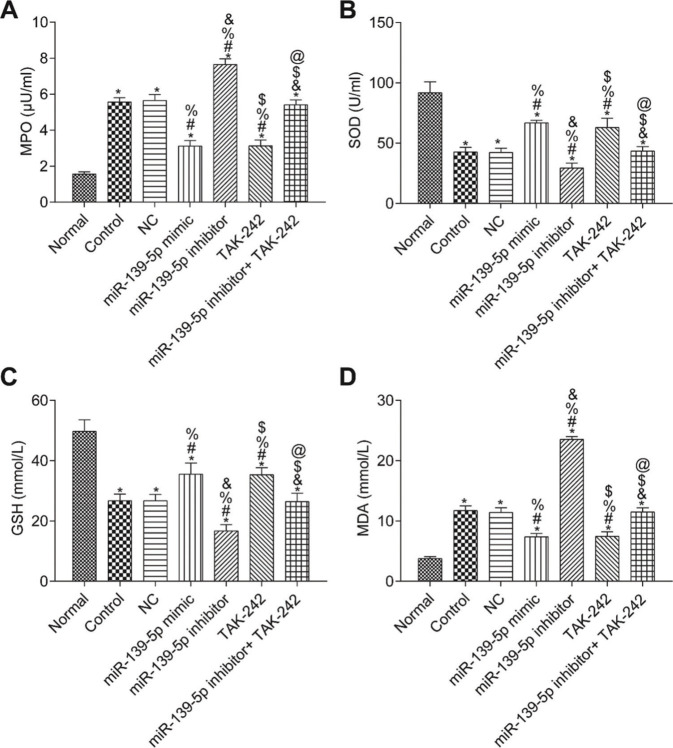
Levels of MPO (A), SOD (B), GSH (C), MDA (D) in the lung tissue of mice in each group. Significant comparisons with each group, where *p*<0.05, are indicated by: “*” for the Normal group, “#” for the Control group, “%” for NC group, “&” for the miR-139-5p mimic group, “$” for the miR-139-5p inhibitor group, and “@” for the TAK-242 group. MPO, myeloperoxidase; SOD, superoxide dismutase; GSH, glutathione; MDA, malondialdehyde.

The measurements of MPO, SOD, GSH, and MDA in the lung tissue of each group are shown in Figure 5. Compared with those in the Normal group, the SOD and GSH contents in the lung tissues of the other groups were significantly lower, while the contents of MPO and MDA were significantly higher (*p*<0.05). Compared with those in the control group, the levels of MPO, SOD, GSH, and MDA were not significantly different in the NC group and miR-139-5p inhibitor+TAK-242 group (*p*>0.05). Moreover, the levels of SOD and GSH were significantly higher, but the levels of MPO and MDA were significantly lower in the miR-139-5p mimic group and TAK-242 group (*p*<0.05), while these indicators exhibited opposite results in the miR-139-5p inhibitor group (*p*<0.05). Compared with those in the miR-139-5p inhibitor group, the levels of SOD and GSH in the miR-139-5p inhibitor+TAK-242 group were significantly higher, and the levels of MPO and MDA were significantly lower (*p*<0.05).

## DISCUSSION

The development of ALI and ARDS is mediated by a variety of intracellular signal transduction pathways (20,21). The TLR4/MyD88/NF-κB signaling pathway plays a role in ALI (22). Activation of NF-κB by bacterial endotoxin is essential for the production of transcriptional and pro-inflammatory mediators, including TNF-α, IL-1β, and IL-6 (22).

We predicted a targeting relationship between MyD88 and miR-139-5p through http://www.targetscan.org, while the dual luciferase reporter assay confirmed that miR-139-5p targets and negatively regulates MyD88. As no study has reported this relationship, we further validated the results in the lung tissue of mice. We injected mice with miR-139-5p mimic and miR-139-5p inhibitor, and the results showed that miR-139-5p overexpression inhibits the the TLR4/MyD88/NF-κB signaling pathway, while silencing miR-139-5p had an opposite effect. Therefore, miR-139-5p targets and inhibits the expression of genes involved in the TLR4/MyD88/NF-κB signaling pathway in septic mice with ALI.

We investigated the effects of TLR4/MyD88/NF-κB pathway regulation by miR-139-5p on ALI in septic mice. In the septic mice, we found that the expression of miR-139-5p was downregulated while that of TLR4, MyD88, and NF-κB p50 was upregulated; thus, we also injected mice with the TLR4 inhibitor, TAK-242, to investigate the effect of TLR4 signaling. The results showed that—comparing the Control group and the miR-139-5p inhibitor group—the inflammatory factor release and oxidative stress-induced injury were significantly decreased, and pulmonary function improved. These results indicate that the inhibition of TLR4 signaling alleviates inflammatory factor release and oxidative stress induced upon the silencing of miR-139-5p and activation of the TLR4/MyD88/NF-κB signaling pathway. Jiang et al. (23) found that polydatin ameliorates lung injury caused by LPS by inhibiting the TLR4/MyD88/NF-κB signaling pathway. Zhang et al. and others have reported similar results (24,25). The results of these studies are consistent with our findings, proving that miR-139-5p targets the MyD88/NF-κB signaling pathway in septic mice, thereby inhibiting inflammatory factor release and oxidative stress-induced damage, thus playing a protective role.

These data are preliminary, and therefore it is necessary to investigate the mechanism by which the TLR4/MyD88/NF-κB pathway plays a role in ALI. Further studies focusing on the use of miR-139-5p will enable the treatment of ALI will be valuable.

## CONCLUSION

In conclusion, miR-139-5p mediates the TLR4/MyD88/NF-κB signaling pathway by targeting the MyD88 gene, thereby inhibiting inflammatory factor release and oxidative stress-induced damage. This may be mechanism underlying the pathogenesis of ALI and provides new insights for the treatment of sepsis-induced ALI.

## AUTHOR CONTRIBUTIONS

Xiuxiu Zhang, Xin Liu, Rui Chang, and Yue Li were involved in study design. Clinical and experimental studies were performed by Xiuxiu Zhang and Xin Liu. Statistical analysis included input from Xiuxiu Zhang, Rui Chang, and Yue Li, while Rui Chang was additionally responsible for data acquisition and analysis. The manuscript was prepared by Xiuxiu Zhang and Yue Li, while manuscript editing was performed by Xiuxiu Zhang and manuscript review by Yue Li. Yue Li was responsible for study concept development, definition of the intellectual content, and was the guarantor of study integrity.

## Figures and Tables

**Table 1:** qRT-PCR primer sequences.

Genes	Sequences
	F:5′-CTCCACTCCTCCCTTTCCTC-3′
miR-139-5p	
	R:5′-GCGGTAAGAAGCAGAGCAG-3′
	F:5′-ACAAACGCCGGAACTTTTCG-3′
TLR4	
	R:5′-GTCGGACACACACAACTTAAG-3′
	F:5′-TTGCCAGCGAGCTAATTGAG-3′
MyD88	
	R:5′-ACAGGCTGAGTGCAAACTTG-3′
	F:5′-TGTCTGCACCTGTTCCAAAG-3′
NF-κB p50	
	R:5′-TCAGCATCAAACTGCAGGTG-3′
	F:5′-CTCGCTTCGGCAGCACA-3′
U6	
	R:5′-AACGCTTCACGAATTTGCGT-3′
	F:5′-TCTCCCTCACAATTTCCATCCC-3′
GAPDH	
	R:5′-TTTTTGTGGGTGCAGCGAAC-3′

TLR4 = toll-like receptor 4. MyD88 = myeloid differentiation factor 88.
